# Convolutional Neural Network for Breast and Thyroid Nodules Diagnosis in Ultrasound Imaging

**DOI:** 10.1155/2020/1763803

**Published:** 2020-01-10

**Authors:** Xiaowen Liang, Jinsui Yu, Jianyi Liao, Zhiyi Chen

**Affiliations:** ^1^Department of Ultrasound Medicine, The Third Affiliated Hospital of Guangzhou Medical University, 63 Duobao Road, Guangzhou, 510000 Guangdong, China; ^2^Laboratory of Ultrasound Medicine and Artificial Intelligence, Liwan Experimental Center, The Liwan Hospital of the Third Affiliated Hospital of Guangzhou Medical University, 35 Liwan Road, Guangzhou, 510000 Guangdong, China

## Abstract

**Objective:**

The incidence of superficial organ diseases has increased rapidly in recent years. New methods such as computer-aided diagnosis (CAD) are widely used to improve diagnostic efficiency. Convolutional neural networks (CNNs) are one of the most popular methods, and further improvements of CNNs should be considered. This paper aims to develop a multiorgan CAD system based on CNNs for classifying both thyroid and breast nodules and investigate the impact of this system on the diagnostic efficiency of different preprocessing approaches.

**Methods:**

The training and validation sets comprised randomly selected thyroid and breast nodule images. The data were subgrouped into 4 models according to the different preprocessing methods (depending on segmentation and the classification method). A prospective data set was selected to verify the clinical value of the CNN model by comparison with ultrasound guidelines. Diagnostic efficiency was assessed based on receiver operating characteristic (ROC) curves.

**Results:**

Among the 4 models, the CNN model using segmented images for classification achieved the best result. For the validation set, the sensitivity, specificity, positive predictive value (PPV), negative predictive value (NPV), accuracy, and area under the curve (AUC) of our CNN model were 84.9%, 69.0%, 62.5%, 88.2%, 75.0%, and 0.769, respectively. There was no statistically significant difference between the CNN model and the ultrasound guidelines. The combination of the two methods achieved superior diagnostic efficiency compared with their use individually.

**Conclusions:**

The study demonstrates the probability, feasibility, and clinical value of CAD in the ultrasound diagnosis of multiple organs. The use of segmented images and classification by the nature of the disease are the main factors responsible for the improvement of the CNN model. Moreover, the combination of the CNN model and ultrasound guidelines results in better diagnostic performance, which will contribute to the improved diagnostic efficiency of CAD systems.

## 1. Introduction

Thyroid and breast diseases are common superficial problems [1, 2]. Epidemiological data show that the five-year survival rates for breast cancer and thyroid carcinoma rank first and second [3, 4], and thus, early diagnosis and treatment favor a good prognosis [5]. Ultrasound is the primary means of detection for thyroid and breast diseases. However, compared with computerized tomography (CT) and magnetic resonance imaging (MRI), the quality of an ultrasound image is more easily influenced by a variety of factors, and the specificity is low in the diagnosis of certain thyroid and breast diseases (such as identifying thyroid adenoma and nodular goiter or identifying sclerosing adenosis of the breasts and breast cancer). Moreover, manually scanning the thyroid and breast with ultrasound is time-consuming and subjective. Considering these limitations, there is a need for new diagnostic strategies.

Progress in artificial intelligence has provided new solutions in the medical field. Artificial intelligence uses symbol-processing theory to find patterns in data at a level to potentially learn, train, and analyze massive amounts of data. Artificial intelligence can quantitatively assess complex subjects and endow machines with human-like thinking. In recent years, the application of artificial intelligence in the medical field has solved many complex medical problems. For ultrasound, artificial intelligence technology is mainly used in image classification, that is, identifying and distinguishing images based on different features extracted from a large number of images, thereby reaching the goal of intelligent diagnosis. Traditional image classification technology establishes a diagnostic classification model by manually extracting and screening features [6, 7]. For instance, Huang et al. extracted 140 texture features of breast nodule images and applied a support vector machine (SVM) algorithm to carry out a classification diagnosis, which obtained better results [8]. However, as the amount of data gradually increases and the required features become increasingly complex, the manual screening of features can no longer satisfy the application demand. In the early 21^st^ century, deep learning based on traditional classifiers was developed to reduce the dimensionality of data (conversion from high-dimensional data to low-dimensional data) through training in a multilayer neural network. Deep learning produces a high-efficiency classification model by carrying out repeated training, learning, and feedback using known data sets to achieve the automatic extraction and screening of classification features. Research has confirmed that the deep learning algorithm is applicable in the establishment of a big data sample model [9]. The classification accuracy of the model can be further improved by image preprocessing (such as image segmentation [10, 11]). The combination of ultrasound with artificial intelligence exploits the objectivity, stability, and accuracy of artificial intelligence to compensate for the subjectivity, operator dependence, and low specificity in thyroid and breast ultrasound diagnosis. It is essential that ultrasound methodology adapts to advances in big data and cloud technology.

Commercial applications of artificial intelligence in superficial ultrasound are emerging, such as ultrasonic computer-aided diagnostic (CAD) software for the thyroid, “Amcad-UT,” which was researched and developed by AmCad BioMed of Taiwan and has been successfully marketed in the United States, the European Union, and China [12]. However, this type of CAD system can only conduct analyses for single-organ diseases, and some of the software can only be loaded on the ultrasound instrument. Part of the images must be output in a Digital Imaging and Communications in Medicine (DICOM) format to carry out the analysis, and this process is not conducive to practical application and more widespread use. In addition, previous studies have indicated an association between the incidence rates of thyroid and breast carcinoma in women, and this association might be related to the effect of estrogen, the transport mechanism of iodine, and screening bias [13, 14]. Consequently, in Asia, departments of “Thyroid Breast Surgery” are common, and sonograms of the thyroid and breast are frequently examined in the same test.

To optimize the cost ratio, thyroid and breast lesions with similar ultrasonic diagnostic features were combined in this paper to establish a thyroid-breast nodule ultrasonic diagnostic system based on deep learning technology. Based on the powerful feature extraction capability of deep learning, this system expands the scope of application to improve the practical application and promotion value of the model. In addition, a comparative assessment of the model was performed through the collection of prospective cases, and the significance of the combined application of the two methods in improving diagnostic efficiency was studied.

The framework of this article is as follows: (1) a multiorgan CAD system based on a convolutional neural network is developed, and the impacts of different preprocessing techniques on diagnostic efficiency are further investigated; (2) the diagnostic efficiencies of the CNN and clinical guidelines are compared and validated.

## 2. Materials and Methods

### 2.1. Subjects and Dataset

In total, 537 ultrasound images from 221 patients in the Picture Archiving and Communication Systems acquired during 2015 to 2018 were retrospectively analyzed (Table 1). The images came from a variety of machines, including Phillips IU22, IE33, or CX50 (Philips Healthcare, Eindhoven, the Netherlands); HITACHI Hi Vision Preirus or Ascendus (Hitachi Ltd, Tokyo, Japan); GE Logiq E9, S6, S8, E6, or E8 (GE Healthcare, Milwaukee, WI); Siemens S1000/S2000 (Siemens Healthineers, Munich, Germany); and Toshiba Aplio 300 or Aplio 500 (Toshiba Medical Systems, Tokyo, Japan). Histopathological examination, including aspiration biopsy and surgery pathology, was used as the reference standard. Images without artifacts were eligible for participation. Nodules larger than the region of interest (ROI), diffuse diseases, and cystic nodules were excluded. Multiple lesions without a separable nodule were excluded as well. Moreover, another set of 85 images from 38 patients were prepared for the initial validation of the CNN-based CAD systems. All images were stored as PNG data. In this study, 30 additional cases were enrolled prospectively to evaluate the value of the separate and combined CAD systems and the ultrasound guidelines. The study was approved by the Ethics Committee of the Third Affiliated Hospital of Guangzhou Medical University.

### 2.2. Ultrasound Examination

Cases were enrolled prospectively according to the principles as follows.

#### 2.2.1. Thyroid Ultrasound Scanning

The patient assumed a supine position with the head leaning slightly backwards, fully exposing the anterior and lateral areas of the neck. The probe was first placed on the thyroid gland laterally to observe the thyroid capsule and parenchymal situation on the transverse section, and then, the probe was rotated 90° and placed on one of the side lobes to longitudinally observe the thyroid capsule and parenchymal situation and to measure the size of the thyroid gland.

#### 2.2.2. Breast Ultrasound Scanning

The patient assumed a supine position with both hands lifted above the head to fully expose both breasts and the axillary areas. The bilateral mammary glands, breast lesions, and bilateral axillary lymph nodes were explored. Quadrant positioning and clock positioning methods were used to clearly locate the lesions.

The observed thyroid and nodule ultrasonic features included size of the nodule, morphology, location, echo, margin, boundary, surrounding tissue, posterior echo, and whether there was calcification. Multiple clear ultrasonic images were taken.

### 2.3. Image Preprocessing

**Figure 1:**
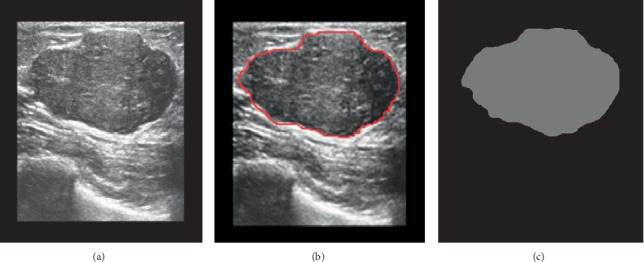
Procedure of lesion boundary drawing. (a) Original image. (b) Targeted lesion region selected by the physician (red contours). (c) The corresponding binary masks used for training the CNN.

Since original ultrasound images (especially for images of low quality) contain large amounts of imprecise and incomplete information, preprocessing is essential for data consistency and accuracy. In this paper, the Windows built-in drawing software was used to standardize the sample images. Centering on the lesion, the images of 537 cases of training sets and 85 cases of validation sets were uniformly cut to 315 × 315 pixels. A red line was used to manually outline the boundary of the lesion in the images after uniformity processing and saved in PNG format as “original image training set” and “original image validation set.” The corresponding binary masks used for training the CNN were separately saved in PNG format as “segmentation training set” and “segmentation validation set” (Figure 1). All image processing was performed by a physician with 4 years of experience in identifying lesion edges, and a senior physician improved the match if the boundary was not well-identified.

### 2.4. Training and Validating the CNN-Based CAD System

The CNNs were developed using DIGITS (Deep Learning GPU Training System, NVIDIA, USA). Inceptive characteristics of GooLeNet and CaffeNet were exploited in classification. We set the image type as “Color,” the base learning rate as 0.001, and the training epochs as 200. Transfer learning was performed to increase the sample size. Finally, the model used 5-fold cross validation for internal assessment.

**Figure 2:**
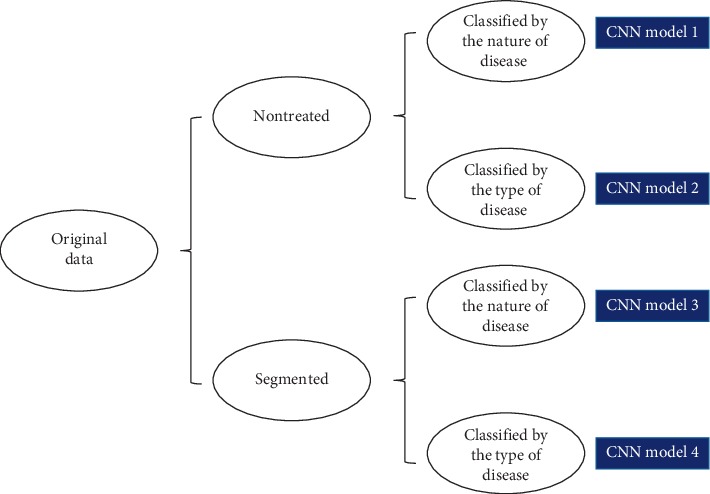
Structures of the 4 CNNs.

We developed CNN models using the original images or segmented images. Each group was classified into two subgroups: one subgroup was classified by the nature of the disease as benign or malignant and the other one was classified by type of disease (fibroadenoma, invasive carcinoma, nodular goiter, and papillary carcinoma). The initial validation of 85 cases was used to verify and choose the outstanding model of the 4 CNN models for the following steps (Figure 2).

### 2.5. Performance Measurement

In this study, diagnosis performance was evaluated by accuracy, sensitivity, and specificity. A total of 140 image samples that met the requirements above were selected prospectively. We tracked the pathological results of all cases. The model was used for automatic identification and classification diagnosis on the prospective validation set to obtain the model diagnosis results. The classification of the 2017 American College of Radiology (ACR) Thyroid Imaging Reporting and Data System (TI-RADS) [15] and the 2013 Breast Imaging Reporting and Data System (BI-RADS) [16, 17] was used to assess the risk of malignancy for thyroid and breast nodules. In the ACR TI-RADS, points are given for all ultrasound features, with more suspicious features being awarded additional points. The point total determines the nodule's level, which ranges from TR1 (benign) to TR5 (high suspicion of malignancy). In the 2013 BI-RADS, nodules are also categorized as category 1 (benign) to 5 (high suspicion of malignant) according to the ultrasound features. Nodules with TR4 and higher or BI-RADS category 4 and above were defined as having a malignant tendency. A senior physician with 15 years of ultrasound experience performed the above evaluation.

### 2.6. Statistical Analysis

The Shapiro–Wilk test was used to analyze the normality of the data. The quantitative data were presented as the means ± SDs or interquartile ranges in case of asymmetry. The sensitivity, specificity, positive predictive value (PPV), negative predictive value (NPV), and accuracy were calculated by comparing the pathological findings. Significant differences between the groups were assessed using the paired McNemer's test and chi-squared test. *P* < 0.05 indicated a statistically significant difference. The diagnostic efficiencies of the CNN, ultrasound guidelines, and their combination were evaluated by receiver operating characteristic (ROC) curves. The statistical analyses were accomplished with SPSS software version 16.0 (SPSS, Chicago, IL).

## 3. Results

### 3.1. Dataset of the CNN System

The 537 images consisted of 158 thyroid nodules and 379 breast nodules, including 287 benign nodules (53.4%) and 250 malignant nodules (46.6%). Since one case of papillary thyroid carcinoma was mismatched, the initial validation set ultimately included 84 cases.

### 3.2. Diagnostic Efficiency of the CNN Systems

**Figure 3:**
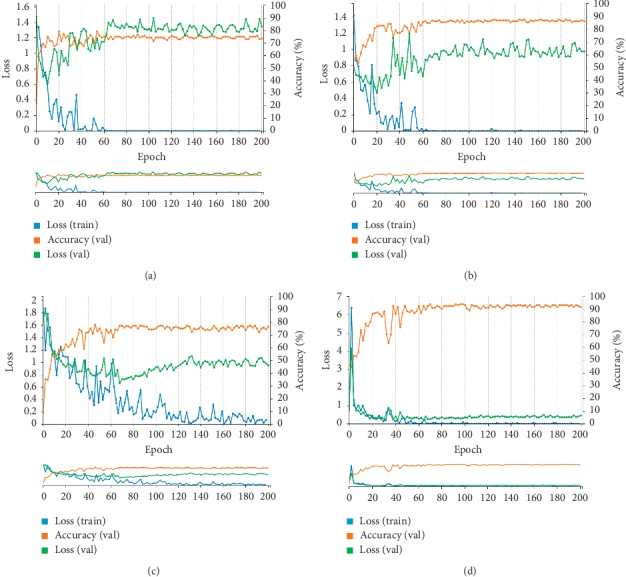
Training set accuracy and loss curves of the CNNs. The orange lines represent the changes in accuracy of the validation set, the blue lines represent the dynamic loss of the training set, and the green lines represent the dynamic loss of the validation set. (a) Nontreated images classified by type of disease. (b) Nontreated images classified by nature of the disease. (c) Segmented images classified by type of disease. (d) Segmented images classified by nature of the disease.

The training curves of the groups with epochs are shown in Figure 3. The training set accuracy of the groups was 75% (nontreated images classified by type of disease), 88% (nontreated images classified by nature of the disease), 78% (segmented images classified by type of disease), and 92% (segmented images classified by nature of the disease). After 200 epochs, the training was stopped due to the absence of further improvement in both accuracy and loss.

**Figure 4:**
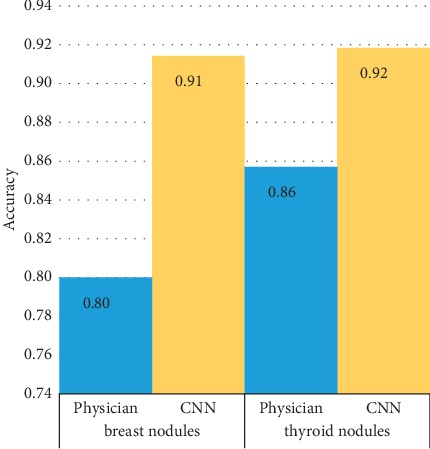
Comparison of diagnostic performance for breast and thyroid nodules.

In addition, Table 2 lists the diagnostic efficiencies of the different CNN models in the initial validation, and Tables 3 and 4 show the diagnostic consistencies of the CNN models and pathology. The results of the CNN model, which were expressed as a percentage, were divided into “Benign tendency” and “Malignant tendency.” The diagnostic criterion was set at “>50%.” The comparison of the results of the different processing methods indicated that after segmentation, fewer benign lesions were misclassified as malignant lesions (18 vs. 0), but the number of cases in which invasive carcinoma was misclassified as papillary carcinoma increased (3 vs. 17). The results indicated that the CNN model developed by segmenting images and classifying by the nature of the disease achieved better diagnostic sensitivity, specificity, and accuracy among the 4 models (*P* < 0.05). According to the validation set, great diagnostic performance was observed for the diagnosis of breast and thyroid nodules (Figure 4). We used this model to perform the subsequent validations.

### 3.3. Diagnostic Efficiency of ACR TI-RADS and BI-RADS Individually and Combined with the CNN System

**Figure 5:**
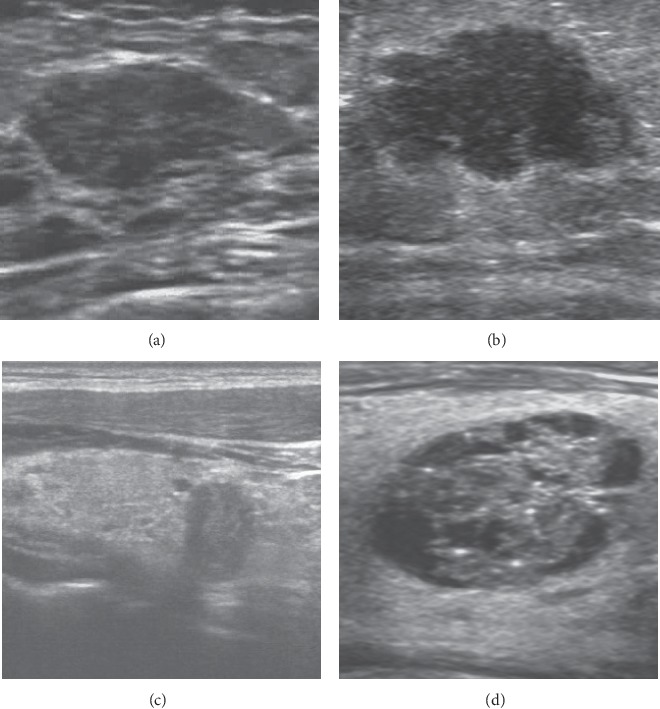
Diagnosis of thyroid and breast nodules with benign or malignant tendency by ultrasound guidelines and CNN. (a) Fibroadenoma. The nodule was classified as BI-RADS 3, while the diagnosis of CNN was benign tendency (93.24%). (b) Invasive breast carcinoma. The nodule was classified as BI-RADS 4, while the diagnosis of CNN was malignant tendency (100%). (c) Papillary thyroid carcinoma. The nodule was classified as TR5, while the diagnosis of CNN was malignant tendency (99.39%). (d) Nodular goiter. The nodule was classified as TR2, while the diagnosis of CNN was benign tendency (100%).

A total of 140 images were analyzed prospectively. The image data were rooted in 14 cases of thyroid nodules (including 12 nodular goiters and 2 papillary carcinoma) and 16 breast nodules (including 7 fibroadenoma and 9 invasive carcinoma). We used 2017 ACR TI-RADS and 2013 BI-RADS as the ultrasound guidelines for the diagnosis of thyroid and breast nodules. According to the ACR TI-RADS categories, 51 images were classified as TR2 to TR3, and 12 images were classified as TR4 to TR5. Among the 77 ultrasound images of breast nodules, 19 images were classified as BI-RADS 2 to 3, while the remaining 58 images were classified as BI-RADS 4 to 5. During the CNN diagnosis, 52 images of thyroid nodules and 16 images of breast nodules were considered to have benign tendency, while another 11 cases of thyroid nodules and 61 cases of breast nodules were considered to have malignant tendency (Figure 5).

**Figure 6:**
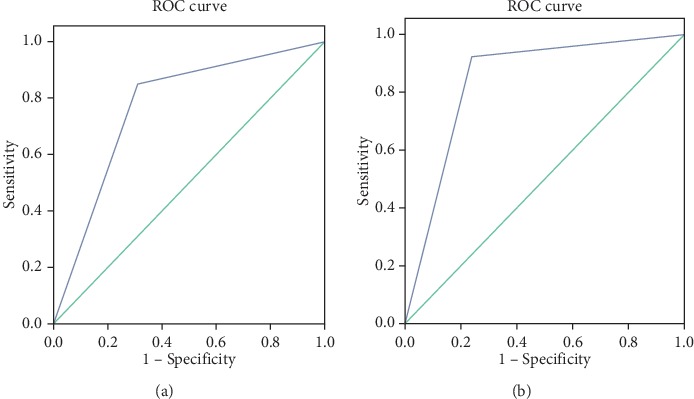
ROC curves of the CNN model and ultrasound guidelines. (a) The AUC of the CNN model was 0.769 (95% CI: 0.688–0.850). (b) The AUC of the ultrasound guidelines was 0.842 (95% CI: 0.773–0.910).

In this study, the performance of the CNN model was inferior to that of the ultrasound guidelines. However, there was no significant difference between the two diagnostic methods in the area under the receiver operating characteristics curve (AUC) (0.769 vs 0.842, *P* > 0.05, Figure 6). Compared with independent use, the combination of CNN and ultrasound guidelines resulted in an improved specificity of 92.5% but lower sensitivity (78.5%). The results of the chi-squared test demonstrated that there was a significant difference between independent use and combination with CNN (*P* < 0.05), with superior diagnostic efficiency of the combination (Table 5).

## 4. Discussion

This study mainly aimed to establish a multiorgan ultrasonic CAD model based on a CNN deep learning algorithm and to validate the clinical value of the model by comparison with the efficiency of ultrasound diagnostic criteria. Although CAD has been broadly applied in CT and MRI, its application in ultrasound images is relatively rare. We believe that screening will be the most common application of artificial intelligence, and further improvements in CNNs (such as multiorgan application) may provide an accurate and efficient new strategy for thyroid and breast cancer screening.

To verify the performance of the different image preprocessing strategies, in this study, we compared the efficiency between the original image and the model established from the image of the extracted nodule pixels after segmentation, mainly focusing on the margin features and the surrounding tissue features. The results showed that the performance of the CNN established by the segmented image was better than that of the original image, and the differences in margin features were enhanced due to the removal of echo interference from the surrounding tissue. After segmentation, the number of misdiagnoses in the identification of malignant and benign nodules clearly decreased (18 vs. 0). In addition, the lack of segmentation and recognition of tissue around the lesion led to an increase in the number of invasive carcinomas misclassified as papillary carcinoma (3 vs. 17). We further compared the performance of the CNN for breast and thyroid nodules with that of the physician, and the results showed that the diagnostic accuracy of CNN was superior for both breast and thyroid nodules (92% for thyroid and 91% for breast). However, for separate diagnostic performance, the accuracy rates decreased to 42.9% for thyroid nodules (22 of the thyroid nodules were correctly diagnosed, while 27 cases were misdiagnosed) and 82.9% for breast tumors (29 cases of breast tumors were correctly diagnosed, while 6 cases were misdiagnosed) in the validation set. The major cause was the lack of segmentation of the surrounding tissue, which led to the misclassification of invasive carcinoma and papillary carcinoma. These results confirmed that compared with the original images, the use of preprocessed ultrasound images can effectively improve the performance for classifying benign and malignant lesions but still has poor efficiency in further differentiating the type of disease [18]. However, once the target organ is confirmed, there will not be a problem in practical application.

Prior studies of CAD systems based on deep learning algorithms have consistently observed high sensitivity and low specificity, especially for studies with small sample sizes. For instance, in a retrospective study that developed a CNN model using 342 cases of thyroid nodules, the sensitivity, specificity, PPV, NPV, accuracy, and AUC of the CNN model were 96.7%, 48.5%, 87.3%, 86.2%, 82.2%, and 0.73, respectively, compared with values of 96.2%, 75.7%, 90.2%, 89.7%, 90.1%, and 0.87, respectively, for 2017 ACR TI-RADS [19]. Another study compared the deep learning system with the Automated Breast Ultrasound (ABUS) based on BI-RADS [20]. Most studies have demonstrated high sensitivity and low specificity of CNN models, similar to our study. According to our CNN model, the sensitivity, specificity, PPV, NPV, accuracy, and AUC were 84.9%, 69.0%, 62.5%, 88.2%, 75.0%, and 0.769, respectively, lower than the values obtained according to the ultrasound guidelines (92.5%, 75.9%, 70.0%, 94.3%, 82.1%, and 0.842, respectively). However, the differences were not significant (*P* > 0.05). In addition to the small sample size, the low specificity is probably due to selection bias and subjectivity of the training set, discordance among the diagnostic standards, and the difficulty of classifying isoechoic nodules or nodules without a clear margin. Despite the above problems, our study confirms the clinical value of the CNN model in ultrasound diagnosis. To further increase the diagnostic efficiency, parameter optimization and big sample data are needed. Additionally, compared with independent use, the combination of CNN and ultrasound guidelines yielded higher specificity (85.1%), supporting the adaptation of multiple diagnostic criteria for CNN models.

The neural network deep learning method is based on a powerful feature recognition function that can, by learning and analyzing a large amount of data, automatically find and extract regular features to achieve good classification and diagnostic results [21–24]. When establishing the neural network deep learning model, it is difficult to adjust the parameters of the model to fix on certain features as targets, and a higher diagnostic accuracy rate can be obtained by repeated training over a long period of time. To address generalization and reduce overfitting to improve the expression of high-level features in CNN models, more data and parameter adjustments are needed [25]. A large sample size is an important guarantee for improving the accuracy of the deep learning network, and studies have confirmed that deep learning has better accuracy than other algorithms (such as SVMs) in large sample data [26, 27]. In addition, drawing the outline of the nodule margin is another factor that affects the classification diagnosis; therefore, more senior physicians should be included in this step to ensure accuracy in manually drawing the outline. Furthermore, to achieve a more objective evaluation of the model, it is necessary to apply the same data set to other algorithmic models for comparison, instead of comparing diagnostic efficiency using different data sets. This is also a limitation of this paper that awaits improvement in further research.

## 5. Conclusion

In this study, we propose a thyroid-breast nodule CAD ultrasonic diagnostic system based on deep learning. The system was shown to be useful in ultrasonic screening of the lesions of two organs. The system not only shortens the examination time but also reduces the physician's examination burden. Furthermore, its cost-effectiveness may help promote the application of CAD in ultrasound imaging examination. Nevertheless, the establishment of an efficient, accurate, and valuable CAD model depends on a large amount of sample, which is a difficult problem for the collection of image data [25]. A multicenter study may help with the development of large-sample research. As the amount of data increases, classifier algorithm parameters further improve, and deep learning technology benefits from supervised and nonsupervised coordination. The accurate extraction of numerous effective classification features will transform the ultrasonic CAD system into the truly “intelligent diagnosis” of our expectations.

## Figures and Tables

**Table 1:** Details of the data studied in our experiments.

	Diseases	Patients	Training set	Validation set
Breast tumors	Fibroadenoma	91	182	22
	Invasive carcinoma	56	197	13

Thyroid nodules	Nodular goiter	52	105	17
	Papillary carcinoma	22	53	33

Total		221	537	85

**Table 2:** Diagnostic performance of the validation set in different CNN models.

	Consistent with pathology	Sensitivity (%)	Specificity (%)	PPV (%)	NPV (%)	Accuracy (%)
Nontreated, classified by type of disease	48	55.6	76.9	73.5	60.0	57.1
Nontreated, classified by nature of the disease	58	77.0	63.2	60.0	79.5	69.0
Segmented, classified by type of disease	50	86.7	84.6	86.7	84.6	59.5
Segmented, classified by nature of the disease	74	81.8	100.0	100.0	74.4	88.1

**Table 3:** Verification of the CNN model classified by type of disease based on segmented images.

		Pathology			
		Nodular goiter	Invasive carcinoma	Papillary carcinoma	Fibroadenoma
CNN model (segmented, classified by type of disease)	Nodular goiter	11	0	4	2
	Invasive carcinoma	0	11	1	1
	Papillary carcinoma	0	17	10	5
	Fibroadenoma	2	0	2	18

**Table 4:** Verification of the CNN model classified by type of disease based on nontreated images.

		Pathology			
		Nodular goiter	Invasive carcinoma	Papillary carcinoma	Fibroadenoma
CNN model (nontreated, classified by type of disease)	Nodular goiter	11	0	3	3
	Invasive carcinoma	1	6	1	5
	Papillary carcinoma	13	3	15	1
	Fibroadenoma	0	5	1	16

**Table 5:** Diagnostic efficiency of the CNN model, ultrasound guidelines and their combination.

	Sensitivity (%)	Specificity (%)	PPV (%)	NPV (%)	Accuracy (%)
CNN model	84.9	69.0	62.5	88.2	75.0
Ultrasound guidelines	92.5	75.9	70.0	94.3	82.1
Combination	77.4	85.1	75.9	86.0	82.1

## Data Availability

All data generated or analyzed during this study are included in this published article.
